# The role of metamemory on cognitive complaints in cancer patients

**DOI:** 10.1002/brb3.1545

**Published:** 2020-03-10

**Authors:** Bénédicte Giffard, Audrey Perrotin, Philippe Allain, Jacques Dayan, Francis Eustache, Jean‐Michel Grellard, Audrey Faveyrial, Florence Joly, Marie Lange

**Affiliations:** ^1^ EPHE INSERM U1077 CHU de Caen Neuropsychologie et Imagerie de la Mémoire Humaine PSL University Normandie Univ UNICAEN Caen France; ^2^ Cancer & Cognition Platform Ligue Contre le Cancer Caen France; ^3^ Laboratoire de Psychologie des Pays de la Loire (LPPL EA 4638) Université d'Angers Université de Nantes Angers France; ^4^ CHGR Pôle Universitaire de Psychiatrie de l'Enfant et l'Adolescent Rennes 1 France; ^5^ Clinical Research Department Centre François Baclesse Caen France; ^6^ Ambulatory Department Centre François Baclesse Caen France; ^7^ INSERM U1086 ANTICIPE Normandie Univ UNICAEN Caen France; ^8^ Medical Oncology Department CHU de Caen Caen France

**Keywords:** cancer patients, cognition, memory, metacognition, metacognitive monitoring, metamemory, neuropsychology

## Abstract

**Objective:**

Although cancer patients frequently report cognitive disturbances, it is commonly asserted a lack of association between cognitive complaints and neuropsychological test performances. Our goal was to better understand the relationships between subjective and objective cognitive scores through a metamemory monitoring assessment.

**Methods:**

Sixty cancer patients currently treated by chemotherapy and/or targeted therapy, and 30 healthy controls (HC) were included. Cognitive complaint was assessed by FACT‐cog, QAM and DEX questionnaires. One or more *z*‐scores ≤−1.65 among these three questionnaires defined the presence of cognitive complaints. Objective cognitive performances assessed episodic memory, processing speed and executive functions/working memory (ESR paradigm, TMT, Stroop, *n*‐back). Metamemory was assessed with a Judgment of Learning (JOL) task.

**Results:**

Patients with cognitive complaints had significantly more depressive and anxiety symptoms (*p*s < .004), and lower performances on several cognitive tests (*p*s < .05) than both patients without complaints and HC. More specifically, analyses of the metamemory scores revealed that HC gave significantly more overestimations (“Yes” judgment and incorrect recall) than patients with cognitive complaints (*p *= .036). For these patients, JOL scores correlated positively with executive functioning (*p*s < .01).

**Conclusion:**

Metamemory monitoring seems to be well‐preserved during cancer. What is more, patients make less overestimation than HC, and they do not underestimate their memory. An accurate self‐estimation of memory abilities in cancer patients, particularly those with mild cognitive deficits, may play an adaptive function. Our results suggest that the discrepancy frequently reported between cognitive complaints and objective cognitive scores may not be related to metamemory monitoring dysfunction.

## Graphical abstract

Cancer patients do not underestimate their memory performance. Cancer patients make less overestimation than healthy controls.

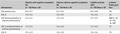

## 1 BACKGROUND

Some non‐central nervous system (non‐CNS) cancer patients report mild cognitive disturbances such as memory problems (Hodgson, Hutchinson, & Wilson, 2013; Root, Andreotti, & Tsu, 2016). These complaints often occur during cancer treatments. They may persist after the end of treatments (Dhillon et al., 2018; Janelsins et al., 2017; Ng et al., 2018) and may follow heterogeneous trajectories between patients: acute, persistent, intermittent, or even absence of cognitive complaints (Ng et al., 2018). Due to the significant impact on the patient's quality of life (Boykoff, Moieni, & Subramanian, 2009; Hutchinson, Hosking, & Kichenadasse, 2012; Lange & Joly, 2017), a growing body of neuropsychological and neuroimaging studies focuses on cognitive impairment in cancer patients (Joly et al., 2015). Nevertheless, considering the frequent discrepancies between subjective reports of cognitive difficulties and objective measures from neuropsychological tests (Bray, Dhillon, & Vardy, 2018; Hutchinson et al., 2012; Pullens, De Vries, & Roukema, 2010), overall cancer patients' cognitive complaints do not seem to be good predictors of their objective performances.

Such lack of association between subjective and objective cognitive disorders may be explained by several factors. Some neuropsychological tests may be insufficiently sensitive to detect subtle to mild cognitive deficits (Ganz et al., 2013; Pullens et al., 2010). Moreover, neuropsychological tests assess cognitive functioning at a point in time, whereas self‐report questionnaires generally carry on broad periods (Hutchinson et al., 2012; Pullens et al., 2010).

Several studies showed that cognitive complaints were more often associated with psychological factors such as anxiety and depression than with neuropsychological test scores (Hutchinson et al., 2012; Pullens et al., 2010). Preexisting personality traits, such as negative affectivity (Hermelink et al., 2010), or psychological distress resulting from stereotype threat (Schagen, Das, & Vermeulen, 2012) was also associated with cognitive complaints. The presence of mood disturbances could interfere with the subjective perception of memory performances and lead to underestimation of one's own performances.

Thereby, a metamemory deficit could be involved in discrepancies between complaints about memory loss and the normal performance in objective memory tests. Metamemory refers to the ability to estimate one's own memory and is broadly defined as individual's knowledge of, monitoring of, and control of one's own learning and memory processes (Nelson & Narens, 1990). Relationships have been suggested between metamemory and executive processes; neuroimaging studies have confirmed the role of prefrontal cortex in metamemory processing (Chua, Schacter, & Sperling, 2009). Thus, cancer patients could underestimate their cognitive/memory performances because of metamemory dysfunction.

Only one study has focused on metamemory monitoring abilities in cancer patients (Collins, Paquet, & Dominelli, 2017). This one was based on the Feeling of Knowing (FOK) procedure which assess, during a memory task, the prediction to recognize nonrecalled items during the recall phase. The FOK metamemory index refers to the comparison between predictions of future and actual recognition scores. Results from this study showed that breast cancer patients had no significantly different performances on FOK index than healthy controls (HC).

The Judgment of Learning (JOL) procedure is based on the same principle than FOK except it involves the learning memory phase. The JOL is measured by asking participants to predict, at learning, future recall performance. Considering the recovery mode (recall for JOL vs. recognition for FOK), the JOL may be more sensitive than the FOK procedure, especially among patients with mild memory difficulties, such as cancer patients, for whom a ceiling effect is often reached in recognition tasks (e.g., De Simone, Perri, & Fadda, 2019).

The main goal of the present study was to determine whether the discrepancy between cognitive complaints and objective cognitive scores frequently observed in cancer patients could reflect metamemory monitoring dysfunction. We hypothesized that patients with cognitive complaints would have a metamemory dysfunction in the sense of an underestimation of their performances, contrary to patients without cognitive complaints and HC.

## 2 METHODS

### 2.1 Participants

Inclusion criteria were patients currently treated for solid cancer or hematological malignancy by chemotherapy and/or targeted therapy. Noninclusion criteria included cancer of CNS or brain metastasis, neurological comorbidities, psychiatric comorbidities, major cognitive disorders, and documented alcohol or drug abuse. Participants who reported a period of formal education <5 years (end of the first school) were not eligible. Cancer patients were recruited from the French Cancer comprehensive Center of Caen from April 2013 to August 2015. A sample of healthy controls (HC) who met the same inclusion (except cancer diagnosis) and exclusion criteria was recruited by community advertisements. HC were age‐, sex‐ and education‐matched to the whole group of patients (*p*s > .10). Ninety participants took part into the study: 60 non‐CNS cancer patients and 30 HC. All participants provided written informed consent for the study, approved by the local ethics committee (2012‐A01340‐43) and registered at http://ClinicalTrials.gov (NCT02212132).

### 2.2 Procedure

Patients were assessed individually in a quiet testing room during hospitalization for chemotherapy and/or targeted therapy. They were evaluated with neuropsychological and metamemory tests and self‐report questionnaires by a graduate neuropsychologist. HC were assessed individually in a quiet testing room at the University of Caen Normandy. They received the same battery than patients except the FACT‐G and the FACIT‐F.

### 2.3 Measures

The *neuropsychological battery* included standardized neuropsychological tests assessing episodic memory, processing speed, and executive functions/working memory (Encoding Storage Retrieval (ESR) paradigm (Eustache, Desgranges, & Lalevee, 1998), Trail Making test (TMT; Reitan, 1958), Stroop (Stroop, 1935), n‐back; Table S1).

*Metamemory monitoring* was assessed with an episodic memory JOL task proposed by Pinon, Allain, and Kefi (2005). This task determines whether the predictions of later recall are or not consistent with the real recall performances (overestimation or underestimation of memory performances). This is a 20 words paired associated learning task that includes immediate and delayed (after 20 min) recalls. Participants were informed that they would be studying 20 pairs of words (half of them were semantically related) for the delayed recall test. At the time of the learning item‐by‐item, they were asked to judge whether or not they would later recall the second element of the paired words when the first was presented after a delay of 20 min. JOLs were given on a Likert‐like scale (0% = definitely will not recall; 20% = 20% sure, 40% = 40% sure, 60% = 60% sure, 80% = 80% sure; 100% = definitely will recall).

JOL accuracy was assessed by comparing the concordance between item‐by‐item prediction during encoding and item‐by‐item recall performance. To do so, all predictions from 0% to 40% (inclusive) were considered as a “No” prediction of recall, and all predictions from 60% to 100% (inclusive) were considered as a “Yes” prediction of recall. In total, there were 4 response possibilities (Table S2): “Yes” prediction and correct recall (JOL A), “Yes” prediction and incorrect recall (JOL B; overestimation of performances), “No” prediction and correct recall (JOL C; underestimation of performances), and “No” prediction and incorrect recall (JOL D).

Goodman–Kruskal gamma correlation (ranges from −1 to +1) was calculated based on JOLs and recall performances. Large positive values correspond to a strong association between JOL judgments and recall performance, values close to zero indicate the absence of association, and negative values designate an inverse relation.

*Patient‐reported outcomes* (PROs) consisted in three self‐report measures of cognitive complaints: the Functional Assessment of Cancer Therapy‐Cognitive Scale (FACT‐Cog, version 3; higher scores reflect few cognitive complaints; four subscales: Perceived Cognitive Impairments [PCI], Impact on Quality of Life [QoL], Comments from Others [Oth], Perceived Cognitive Abilities [PCA]; Joly et al., 2012; Wagner, Sweet, & Butt, 2009), the memory self‐evaluation questionnaire (QAM; Van der Linden, Wijns, Von Frenkell, Coyette, & Seron, 1989; higher scores reflect high memory complaints) and the Dysexecutive questionnaire (DEX; Bennett, Ong, & Ponsford, 2005; higher scores reflect high dysexecutive complaints).

Depression (Center for Epidemiologic Studies Depression Scale [CES‐D; Radloff, 1977]), anxiety (Spielberger State‐Trait Anxiety Inventory—STAI; Spielberger, 1983), fatigue (Functional Assessment of Chronic Illness Therapy‐Fatigue—FACIT‐Fatigue, version 4; Yellen, Cella, & Webster, 1997), quality of life (FACT Scale‐General—FACT‐G, version 4; Cella et al., 1993), and self‐representations (QRS, scores of certainty and valence; Morel et al., 2015) were also assessed.

*Clinical variables* were cancer type, metastatic status, cancer treatments, medications with potential impact on cognition (Level 3 on the WHO analgesic ladder, anxiolytics, antidepressant treatments, and hypnotics), and psychological support or supportive care.

### 2.4 Statistical analysis

Patients' raw scores were standardized to performance of the HC group. We defined patients “with” cognitive complaints those with at least one *z*‐score ≤−1.65 among PCI, PCA (FACT‐Cog), QAM, or DEX. Patients with a *z*‐score >−1.65 on these scales were classified as “without” cognitive complaints (their *z*‐scores were all between −1 and + 1). Student's *t* tests, ANOVAs (Tukey post hoc), and ANCOVAs were used to compare the scores of the groups (*α* = 5%). The correlations between JOL predictions and other scores were assessed with Spearman's rank correlation coefficient. Given the large number of correlations performed, a *p* value < .01 was considered in order to minimize type I error. All statistical analyses were conducted using STATISTICA (StatSoft, 2013).

## 3 RESULTS

### 3.1 Demographic and clinical characteristics

Sixty cancer patients (54 ± 9 years old) currently treated for cancer, and 30 sex‐ and education‐matched HC (51 ± 7 years old) were included. Patients were mainly women (*n* = 57, 95%) treated for breast cancer (*n* = 50, 83%). All patients were being treated by chemotherapy or targeted therapy.

According to cognitive complaints scores detailed above, 30 patients had cognitive complaints [A] and 30 were patients without cognitive complaint [B] (see Table 1).

**Table 1:** Demographic, clinical characteristics, and patient‐reported outcomes of the participants

Demographic	Patients with cognitive complaints [A] (*n* = 30)	Patients without cognitive complaints [B] (*n* = 30)	Healthy controls [HC] (*n* = 30)	Statistics *χ* ^2^/*F*/*t*	*p* Value (Tukey post hoc)
Female, *n *(%)	28 (93)	29 (97)	26 (87)	*χ* ^2^ = 2.2	.34
Age (years), Mean ± *SD* [range]	56 ± 8 [41–70]	52 ± 10 [31–68]	51 ± 7 [37–63]	*F*_2, 87_ = 3.2	**.045 ** (A = B; **A < HC**; B = HC)
Education level, years of school, Mean ± *SD* [range]	12 ± 3 [9–19]	13 ± 2 [9–18]	13 ± 4 [5–23]	*F*_2, 84_ = 0.5	.59
Clinical					
Cancer: breast, *n *(%)	24 (80)	26 (87)	NA	*χ* ^2^ = 1.18	.27
Cancer with metastasis, *n *(%)	12 (40)	9 (30)		*χ* ^2^ = 0.66	.42
Cancer treatments, *n *(%)					
Surgery	27 (90)	24 (80)		*χ* ^2^ = 1.18	.28
Radiotherapy	10 (33)	10 (33)		*χ* ^2^ = 0	1.0
Chemotherapy ± targeted therapy	30 (100)	30 (100)		*χ* ^2^ = 0	1.0
Nb of line, metastatic treatment, Mean ± *SD*	1.1 ± 1.4	0.9 ± 1.6		*U = 393*	.40
FEC	21 (70)	24 (80)		*χ* ^2^ = 0.8	.37
Taxotere	16 (53)	20 (67)		*χ* ^2^ = 1.1	.29
Herceptin	6 (20)	9 (30)		*χ* ^2^ = 0.8	.37
Hormone therapy	6 (20)	7 (23)		*χ* ^2^ = 0.1	.75
Medications with potential impact on cognition, *n *(%)^a^	11/24 (46)	10/22 (45)	None	*χ* ^2^ = 0	.98
Psychological support or supportive care, *n *(%)	4 (13)	2 (7)	NK	*χ* ^2^ = 0.7	.39
Patient‐reported outcomes, Mean ± *SD*					
Cognitive complaints					
FACT‐Cog‐PCI	37.5 ± 11.0	61.0 ± 6.6	61.4 ± 6.6	*F*_2, 87_ = 82.4	**<.001 **(**A **< (B = HC))
FACT‐Cog‐PCA	12.1 ± 4.0	20.3 ± 2.8	20.9 ± 5.8	*F*_2, 87_ = 38.0	**<.001 **(**A **< (B = HC))
FACT‐Cog‐Oth	14.3 ± 2.2	15.4 ± 0.7	15.7 ± 0.7	*F*_2, 87_ = 8.0	**<.001 **(**A **< (B = HC))
FACT‐Cog‐QoL	8.4 ± 4.0	12.3 ± 4.0	14.3 ± 2.2	*F*_2, 87_ = 21.3	**<.001** (**A **< (B = HC))
QAM	2.7 ± 0.4	2.2 ± 0.3	2.0 ± 0.4	*F*_2, 86_ = 29.8	**<.001** (**A **> (B = HC))
DEX	23.6 ± 11.3	18.0 ± 5.5	19.1 ± 7.9	*F*_2, 87_ = 73.3	**.032** (**A > B**; A = HC; B = HC)
Depression: CES‐D	21.2 ± 10.1	11.5 ± 6.6	9.8 ± 7.4	*F*_2, 87_ = 16.8	**<.001** (**A > **(B = HC))
Anxiety: STAI State	40.2 ± 13.2	33.2 ± 9.9	30.9 ± 8.6	*F*_2, 86_ = 5.9	**.004** (**A **> (B = HC))
Anxiety: STAI Trait	45.2 ± 10.2	36.6 ± 8.9	36.5 ± 8.4	*F*_2, 86_ *= *8.7	**<.001** (**A > **(B = HC))
Fatigue: FACIT‐Fatigue	29.1 ± 11.0	36.2 ± 9.4	NA	*F*_1, 58_ = 7.2	**.010**
Quality of life: FACT‐G					
Total score	69.9 ± 15.4	79.8 ± 11.0	NA	*t*_(58) _= 2.9	**.006**
PWB	19.5 ± 5.0	22.1 ± 3.7		*t*_(58)_ = 2.3	**.023**
SWB	20.0 ± 4.4	21.0 ± 5.5		*t*_(58)_ = 0.8	.43
EWB	16.3 ± 4.7	18.1 ± 4.2		*t*_(58)_ = 1.5	.14
FWB	14.1 ± 5.8	18.7 ± 3.9		*t*_(58)_ = 3.6	**<.001**
Self‐representations: QRS^b^					
Certainty (%)	48.4 ± 18.0	46.7 ± 12.3	49.4 ± 17.0	*F*_2, 87 _= 0.2	.80
Valence (%)	65.8 ± 10.9	68.7 ± 8.2	70.0 ± 8.2	*F*_2, 87 _= 1.6	.21

Abbreviations: DEX, Dysexecutive questionnaire; EWB, emotional well‐being; FACT‐Cog, Functional Assessment of Cancer Therapy‐Cognitive Scale; FWB, functional well‐being; NA, not applicable; NK, not known; Oth, Comments from Others; PCA, Perceived Cognitive Abilities; PCI, Perceived Cognitive Impairments; PWB, physical well‐being; QAM, Memory self‐evaluation questionnaire; QoL, Impact on Quality of Life; QRS, Questionnaire of Self‐Representations; SWB, social/family well‐being.

a Level 3 on the WHO analgesic ladder, anxiolytics, antidepressant treatments, and hypnotics.

b QRS: Certainty/valence score: higher is the score, the more certain/positive the self‐representation is.

Bold values represent significant differences.

The three groups ([A] [B] [HC]) did not differ for gender and education level, but patients with cognitive complaints [A] were significantly older than [HC] (*p* = .048). The two patient groups were not significantly different on age, education level, cancer localization, metastatic status, cancer treatments, medications with potential impact on cognition, and psychological support or supportive care.

### 3.2 PRO results

All cognitive complaint scores were significantly lower for group [A] than for group [B] and [HC] (*p*s < .032; see Table 1).

Anxiety and depressive symptoms were also significantly higher for group [A] than for group [B] and [HC] (*p*s < .004). Group [A] reported more fatigue and had lower quality of life than group [B] (*p*s < .01). No significant difference was found between the three groups for self‐representations scores.

### 3.3 Neuropsychological outcomes

Patients group [A] had significantly lower performances on ESR retrieval, TMT A, TMT B‐A, and Stroop (color, word, and interference) time than patients group [B] and/or [HC] (*p*s < .048; see Table 2). ANCOVAs showed that the effect of group remained significant on ESR retrieval and on Stroop (color and word) after controlling for age, anxiety, and depression. No significant difference between groups was found for ESR encoding, immediate, and delayed recalls (on JOL task), perseverative errors on TMT B, Stroop interference errors, and *n*‐back.

**Table 2:** Neuropsychological performances in the three groups

	Patients with cognitive complaints [A] (*n* = 30)		Patients without cognitive complaints [B] (*n* = 30)		Healthy controls [HC] (*n* = 30)		*p*‐Values (Tukey post hoc)
	Raw scores Mean ± *SD*	Impairment *n *(%)	Raw scores Mean ± *SD*	Impairment *n *(%)	Raw scores Mean ± *SD*	Impairment *n *(%)	
Episodic memory							
ESR, encoding score	12.8 ± 2.2	3 (10%)	13.2 ± 2.5	6 (20%)	13.9 ± 1.8	4 (13.3%)	.11
ESR, retrieval score	8.5 ± 1.7	4 (13%)	9.8 ± 2.1	3 (10%)	9.8 ± 1.8	2 (7%)	**.009 A < (**B = HC**)**
JOL, immediate recall	12.0 ± 4.1	0	12.7 ± 2.7	0	10.9 ± 3.4	2 (7%)	.12
JOL, delayed recall	11.3 ± 4.1	0	12.4 ± 3.3	0	10.5 ± 3.5	2 (7%)	.14
Processing speed							
TMT A, time	38.4 ± 13.1	2 (7%)	31.1 ± 10.1	0	32.7 ± 10.8	1 (3%)	**.038 A > B**; A = HC; B = HC
Stroop, color, time	69.3 ± 16.3	2 (7%)	60.7 ± 11.3	0	60.1 ± 9.6	0	**.01 A > **(B = HC)
Stroop, word, time	49.5 ± 10.4	7 (23%)	44.0 ± 5.5	0	43.0 ± 7.1	0	**.004 A > **(B = HC)
Executive function/working memory							
TMT B‐A, time	63.0 ± 57.2	4/29 (14%)	44.4 ± 26.6	3 (10%)	38.9 ± 20.2	1/28 (4%)	**.048 **A = B; **A > HC**; B = HC
TMT B, perseverative errors	0.4 ± 1.0	2/29 (7%)	0.2 ± 0.5	1 (3%)	0.3 ± 0.7	1/28 (4%)	.45
Stroop, interference, time	56.4 ± 26.2	10 (33.3%)	49.3 ± 18.5	5 (16.7%)	41.3 ± 12.0	1 (3.33%)	**.012 **A = B; **A > HC**; B = HC
Stroop, interference, errors	0.6 ± 1.2	5 (16.7%)	0.2 ± 0.5	1 (3.6%)	0.3 ± 0.6	2 (6.7%)	.14
*n*‐back	45.0 ± 5.3	3/27 (11%)	45.8 ± 2.6	1 (3%)	46.3 ± 2.3	2 (7%)	.37

Note

Impairment rate: cognitive score considered as impaired if ≤−2*SD* of the HC group (Wefel, Vardy, Ahles, & Schagen, 2011).

Abbreviations: NA, not applicable; NK, not known.

Bold values represent significant differences.

### 3.4 Metamemory outcomes

No significant difference was found between the three groups for the JOL gamma score [*F*(2, 86) = 1.39; *p* = .28], indicating globally no difference in the ability to estimate one's own memory (see Table 3).

**Table 3:** Metamemory performances in the three groups of participants

Metamemory	Patients with cognitive complaints [A] (*n* = 30) Mean ± *SD*	Patients without cognitive complaints [B] (*n* = 30) Mean ± *SD*	Healthy controls [HC] (*n* = 30) Mean ± *SD*	*p*‐Values (Tukey post hoc)
JOL gamma score	0.3 ± 0.7	0.5 ± 0.6	0.2 ± 0.8	.28
JOL A	7.2 ± 4.4	9.2 ± 3.9	7.3 ± 4.5	.13
JOL B (overestimation of memory performance)	1.6 ± 2.1	1.9 ± 2.4	3.7 ± 4.5	**.027 **A = B; B = HC; **A < HC**
JOL C (underestimation of memory performance)	7.3 ± 3.5	5.6 ± 3.6	5.7 ± 4.5	.17
JOL D	3.9 ± 2.3	3.3 ± 3.0	3.3 ± 3.6	.70

Note

JOL A: “Yes” judgment and correct recall; JOL B: “Yes” judgment and incorrect recall; JOL C: “No” judgment and correct recall; JOL D: “No” judgment and incorrect recall.

Bold values represent significant differences.

To understand whether participants under‐ or overestimate their recall ability, we examined the proportion of correct and incorrect predictions for each “Yes”/”No” JOL (Table S2). A main group effect was found only for JOL B answer (“Yes” judgment and incorrect recall, *that is,* overestimation of the performances): HC made significantly more overestimations compared with group [A] (*p* = .036), but not to group [B] (*p* = .08). This pattern was maintained when age, anxiety, or depression was controlled. No further significant difference was found between the 3 groups for the other patterns of answers, including JOL C answer (“No” judgment and correct recall), suggesting an absence of underestimation of memory performances in cancer patients whatever their memory complaints are.

### 3.5 Relations between JOL predictions and other measures

Within each group, JOL gamma and JOL B were not significantly associated to anxiety, depression, fatigue, and self‐representations (Table S3).

In group [A], JOL gamma was significantly and negatively associated with TMT B‐A time, and positively associated with *n*‐back scores (*p*s < .008), suggesting that a high metamnesic accuracy is related to high executive functions and working memory scores. In this same group, JOL B prediction was significantly and positively associated with TMT A and TMT B‐A times, TMT B perseverative errors, and Stroop interference errors, and negatively associated with *n*‐back scores (*p*s < .009), suggesting that the overestimation of memory is related to low executive functions and working memory.

In groups [B] and [HC], there was no significant correlation between JOL predictions and other measures (excepted for HC between JOL gamma and JOL recalls, *p*s < .005).

## 4 CONCLUSIONS

This is the first study of metamemory monitoring functioning using a JOL task in cancer patients treated by chemotherapy and/or targeted therapy. The group of patients with cognitive complaints [A] scored significantly lower on neuropsychological scores than patients group without cognitive complaints [B] and HC. Group [A] showed however no deficit on the metamemory monitoring index when compared to the other groups. Instead, they made less overestimation of performances (i.e., lower JOL B answer: “Yes” judgment and incorrect recall) than HC which suggests a better estimation of their memory capacities. Therefore, these results cannot confirm that the discrepancy frequently observed in other studies between cognitive complaints and objective cognitive scores reflects metamemory dysfunction.

Beyond several factors previously described to explain the lack of association between subjective and objective measures (relations with psychological factors and methodological concerns), this discrepancy in some previous studies could in part be due to the heterogeneity of cognitive complaints in cancer patients (Ng et al., 2018) which was not sufficiently taken into account. Contrary to most previous studies using a correlational approach, we chose to divide the patients' group in two subgroups in order to isolate patients without any cognitive complaints, and with a strong homogeneity on cognitive complaint scores. This strong homogeneity may contribute to observe more clear‐cut results than in other studies. Moreover, the comprehensive neuropsychological workup used in this study which includes sensible tests (e.g., ESR) was able to reveal and quantify subtle cognitive changes.

Based on a FOK procedure (accuracy in predicting ability to recognize unrecalled target words), the Collins and colleagues study (Collins et al., 2017) is the only one previously published which assessed metamemory in cancer. In this one, the heterogeneity of cognitive complaints was not taken into account with only one group of patients. Patients had no significantly different performances on FOK metamemory index than HC, suggesting that patients had not deficit in metacognition that could explain discrepancy between subjective and objective cognitive scores. Nevertheless, in the present study, thanks to the JOL accuracy measures (comparing item‐by‐item predictions with item‐by‐item recall performances), we were able to reveal that patients with cognitive complaints [A] produced less overestimation of their performances (less JOL B answer) than HC, suggesting a more accurate estimation of their memory capacities than HC. Besides, the patients, with or without cognitive complaints, did not produce significantly more underestimation of the performances (JOL C answers) than HC. We supposed that, probably due to the recall task related to the JOL (while the FOK is based on a recognition task), JOL measures would be more sensitive than FOK ones to detect significant difference on some metamemory monitoring measures, especially among patients with mild memory difficulties such as cancer patients, but in our study using JOL measures, we have not either identify metamemory deficits in cancer patients. Thereby, in addition to the FOK, the JOL procedure is interesting to explain and to determine whether the discrepancy between cognitive complaints and objective cognitive scores could reflect metamemory dysfunction.

Our results do not support the hypothesis that patients with cognitive complaints would have a metamemory dysfunction in the sense of an underestimation of their performances. Instead, they seem to have a better estimation of their memory capacities than HC. Patients with subtle or mild cognitive impairment may be particularly sensitive to cognitive changes, to feel and evaluate these changes with a high degree of precision. In this study, these patients expressed a complaint that appears to closely reflect their cognitive decline. However, when the analyses controlled for age, anxiety, and depression, some of the significant differences on neuropsychological test performances disappeared between patients with cognitive complaints [A] and without cognitive complaints [B] or HC.

In general population, many other studies exploring memory of associated pairs (Koriat & Bjork, 2005) have shown an overconfidence in memory abilities in healthy subjects. JOLs are best calibrated with actual performance when subjects can make delayed JOLs. This may be due to their ability to experiment their true actual memory (Kimball & Metcalfe, 2003). This is the very case of patients with cancer and cognitive deficits that have experiment their own deficits and may even complain about it. Efklides (2009) underlines that metacognitive experiences offer awareness that links the present with the past learning experiences. This may contribute to explain the reduction of overestimation, particularly when exist both memory complaints and actual memory deficits.

Regarding objective cognitive scores, group [A] had significantly lower performances mainly on processing speed and episodic memory retrieval than group [B] and [HC]. JOL B prediction was significantly associated with several executive function scores, only in group [A]: patients with higher level of executive functioning (flexibility, inhibition) tended to less overestimate their memory performance. Indeed, executive functions are involved in metamemory as showed in studies in patients with executive impairment (Le Berre et al., 2010). Neuroimaging studies have confirmed the role of the prefrontal cortex, involved in executive functioning, in metamemory functioning (Chua et al., 2009).

Patients group [A] reported more fatigue, anxiety, and depressive symptoms than group [B] and HC. Nevertheless, JOL predictions were not related to psychological factors or fatigue, whatever the group.

### 4.1 Clinical implications

Despite frequent discrepancy between cognitive complaints and objective cognitive scores, these measures are complementary. Patients' cognitive complaints should not be minimized and cognitive difficulties should be properly investigated and taken into account to facilitate work resumption of young patients, avoid potential repercussions on autonomy in older patients or on adherence to oral treatments. Psychological factors should be systematically assessed in patients with cognitive complaints to better identify the origin of the difficulties and potentially propose specific care (Lange & Joly, 2017). Examining mechanisms of memory complaints in cancer patients, and precisely metamemory, has both clinical and theoretical interest, and insight into the characterization of their memory dysfunction could help draw up targeted rehabilitation programs. Thereby, considering their well‐preserved metamemory functions as shown in the present study, programs of cognitive training may be effective and should be encouraged, as those programs largely depend on preserved metacognition. Besides, a recent feasibility study suggests that metacognitive strategy training could have positive effect on objective and subjective cognitive performances and quality of life in breast cancer patients after chemotherapy (Wolf et al., 2016).

### 4.2 Study limitations

Patient sample of this study was heterogeneous according to cancer type, metastatic status, and cancer treatments. Furthermore, considering the a posteriori distribution, the three groups of the present study were not matched on age and trait anxiety, but these factors were considered as cofactors for metamemory results and showed almost the same results. Finally, this study was cross‐sectional and did not include assessment before cancer treatments. Further studies with a larger sample and including pre‐ and post‐treatment assessments are encouraged.

## CONFLICT OF INTEREST

Authors have no conflict of interest.

## Data Availability

The data that support the findings of this study are available on request from the corresponding author. The data are not publicly available due to privacy or ethical restrictions.
