# Supplementary material for: The role of metamemory on cognitive complaints in cancer patients
Source: Brain Behav. 2020 Mar 10;10(4):e01545. doi: 10.1002/brb3.1545 (PMC7177574; doi:10.1002/brb3.1545)
Supplement: Supplementary file 1 [file BRB3-10-e01545-s001.docx]

**Supplementary materials**

**Table S1.** Neuropsychological and metamemory tests used

| **Cognitive domain** | **Test** | **Outcome measure** | **Range** |
| --- | --- | --- | --- |
| **Episodic memory** | ESR paradigm [26]: encoding abilities  retrieval abilities  JOL task [29] | Superficial encoding - Recognition  Deep encoding - Free recall  Immediate recall  Delayed recall | [0-16]  [0-16]  [0-20]  [0-20] |
| **Processing speed** | TMT [27]  Stroop [28] | Time to complete part A  Time to complete color card  Time to complete word card | ≥0  ≥0  ≥0 |
| **Executive functions**  **Working memory** | TMT [27]  Stroop (interference) [28]  n-back | Time to complete part B – time to complete part A  Perseverative errors  Time to complete: color-word card – color card  Number of non-corrected errors: color-word card – color card  Number of correct answers | ≥0  ≥0  ≥0  ≥0  [0-48] |
| **Metamemory** | JOL task [29] | Gamma score  ‘Yes’ judgment and correct recall (JOL A)  ‘Yes’ judgment and incorrect recall (JOL B)  ‘No’ judgment and correct recall (JOL C)  ‘No’ judgment and incorrect recall (JOL D) | [-1 - +1]  [0-20]  [0-20]  [0-20]  [0-20] |

ESR: Encoding Storage Retrieval, TMT: Trail Making test, JOL: Judgments-of-learning

**Table S2.** Correspondences between prediction and actual recall performance in a JOL task.

|  | | Item-by-item recall performance | |
| --- | --- | --- | --- |
|  |  | Correct recall | Incorrect recall |
| Item-by-item prediction (JOL) ^†^ | ‘Yes’ judgment (future recall of targeted item) | JOL A | JOL B  (overestimation of memory performance) |
|  | ‘No’ judgment (no future recall of targeted item) | JOL C  (underestimation of memory performance) | JOL D |

^†^ Participants are asked to judge whether or not they will later recall an item

**Table S3**. Correlations between metamemory measures and other variables for the 3 groups

|  | **Variables** | **Groups** | | | | | |
| --- | --- | --- | --- | --- | --- | --- | --- |
|  |  | **Patients with cognitive complaints [A]** | | **Patients without cognitive complaints [B]** | | **Healthy controls**  **[HC]** | |
|  |  | **r** | ***p*** | **r** | ***p*** | **r** | ***p*** |
| **JOL Gamma** | Anxiety - State | -0. 15 | *0.52* | 0.14 | *0.51* | -0.07 | *0.78* |
|  | Anxiety - Trait | -0.34 | *0.12* | -0.08 | *0.70* | -0.25 | *0.33* |
|  | Depression | -0.47 | *0.026* | -0.01 | *0.96* | -0.32 | *0.21* |
|  | Fatigue | -0.12 | *0.58* | -0.26 | *0.19* | NA | *NA* |
|  | QRS – Certainty | 0.10 | *0.60* | 0.20 | *0.33* | -0.19 | *0.43* |
|  | QRS – Valence | 0.31 | *0.11* | 0.29 | *0.16* | 0.11 | *0.66* |
|  | ESR, encoding score | 0.21 | *0.36* | -0.15 | *0.48* | 0.24 | *0.35* |
|  | ESR, retrieval score | 0.45 | *0.034* | -0.20 | *0.34* | 0.21 | *0.43* |
|  | TMT A, time | -0.49 | *0.02* | 0.41 | *0.044* | 0.18 | *0.49* |
|  | Stroop, time color card | -0.42 | *0.051* | 0.31 | *0.15* | 0.10 | *0.69* |
|  | Stroop, time word card | -0.50 | *0.018* | 0.05 | *0.81* | 0.39 | *0.12* |
|  | TMT B – TMT A, time | **-0.57** | ***0.006*** | 0.19 | *0.38* | 0.01 | *0.98* |
|  | TMT B, perseverative errors | -0.30 | *0.17* | -0.12 | *0.59* | -0.23 | *0.37* |
|  | n-back | **0.55** | ***0.008*** | -0.20 | *0.35* | 0.03 | *0.89* |
|  | Stroop interference, time | 0.48 | *0.025* | 0.37 | *0.075* | 0.37 | *0.15* |
|  | Stroop interference, errors | -0.29 | *0.19* | -0.13 | *0.53* | -0.08 | *0.77* |
| **JOL B**  (‘Yes’ judgment; incorrect recall) | Anxiety - State | 0.02 | *0.92* | -0.10 | *0.64* | -0.19 | *0.46* |
|  | Anxiety - Trait | 0.26 | *0.24* | 0.19 | *0.38* | 0.01 | *0.97* |
|  | Depression | 0.20 | *0.37* | 0.06 | *0.80* | -0.04 | *0.89* |
|  | Fatigue | 0.24 | *0.22* | 0.17 | *0.41* | NA | *NA* |
|  | QSR – Certainty | 0.07 | *0.71* | -0.11 | *0.60* | 0.39 | *0.10* |
|  | QSR – Valence | -0.18 | *0.36* | -0.20 | *0.33* | -0.01 | *0.98* |
|  | ESR, encoding score | -0.48 | *0.024* | 0.10 | *0.65* | -0.37 | *0.14* |
|  | ESR, retrieval score | -0.50 | *0.018* | 0.03 | *0.87* | -0.23 | *0.38* |
|  | TMT A, time | **0.70** | ***<0.0001*** | 0.41 | *0.045* | 0.12 | *0.64* |
|  | Stroop, time color card | 0.18 | *0.41* | 0.30 | *0.15* | -0.51 | *0.04* |
|  | Stroop, time word card | 0.32 | *0.15* | 0.50 | *0.81* | -0.29 | *0.26* |
|  | TMT B – TMT A, time | **0.83** | ***<0.0001*** | 0.19 | *0.38* | 0.25 | *0.34* |
|  | TMT B, perseverative errors | **0.68** | ***0.001*** | -0.12 | *0.59* | 0.03 | *0.90* |
|  | n-back | **-0.82** | ***<0.0001*** | -0.20 | *0.35* | -0.21 | *0.41* |
|  | Stroop interference, time | 0.49 | *0.021* | 0.37 | *0.08* | -0.30 | *0.24* |
|  | Stroop interference, errors | **0.55** | ***0.009*** | -0.13 | *0.53* | 0.27 | *0.30* |

QSR: Questionnaire of Self-Representations, ESR: Encoding Storage Retrieval, JOL: Judgement of Learning, TMT: Trail Making Test, PCI: Perceived Cognitive Impairments, PCA: Perceived Cognitive Abilities, QAM: memory self-evaluation questionnaire, DEX: Dysexecutive questionnaire

Results in bold: *p*<0.01
